# 8th German Conference on Chemoinformatics

**DOI:** 10.1186/1758-2946-5-S1-A1

**Published:** 2013-03-22

**Authors:** Uli Fechner

**Affiliations:** 1 GDCh-CIC Division Board Member, Beilstein-Institut zur Förderung der Chemischen Wissenschaften, Trakehner Str. 7-9, 60487 Frankfurt, Germany

## 

The 8th German Conference on Chemoinformatics (GCC2012) was held from the 11^th ^to the 13^th ^of November 2012 in Goslar, Germany and addressed a broad range of current research topics in the realm of computers and chemistry. The CIC division of the Gernan Chemical Society (GDCh) [1] invited the chemoinformatics and molecular modelling community to the GCC2012 to discuss trends and recent developments in the fields of

• Chemoinformatics and Drug Discovery

• Chemical Information, Patents and Databases

• Molecular Modelling

• Computational Materials Science and Nanotechnology

As always, researchers from other research areas of Computational Chemistry were also encouraged to submit contributions. The Scientific Advisory Board compiled an interesting program that consisted of 22 lectures and 53 poster presentations. More than 130 scientists from 20 nations attended the GCC 2012. The large number of attendees from countries other than Germany demonstrates that the conference is an internationally well-established event in the global Chemoinformatics and Modelling community. Traditionally, the conference was opened by the "Free-Software-Session". This session provided room for presentations of as well as discussions about Open-Source software and attracted an audience of both developers and users.

**Figure 1:**
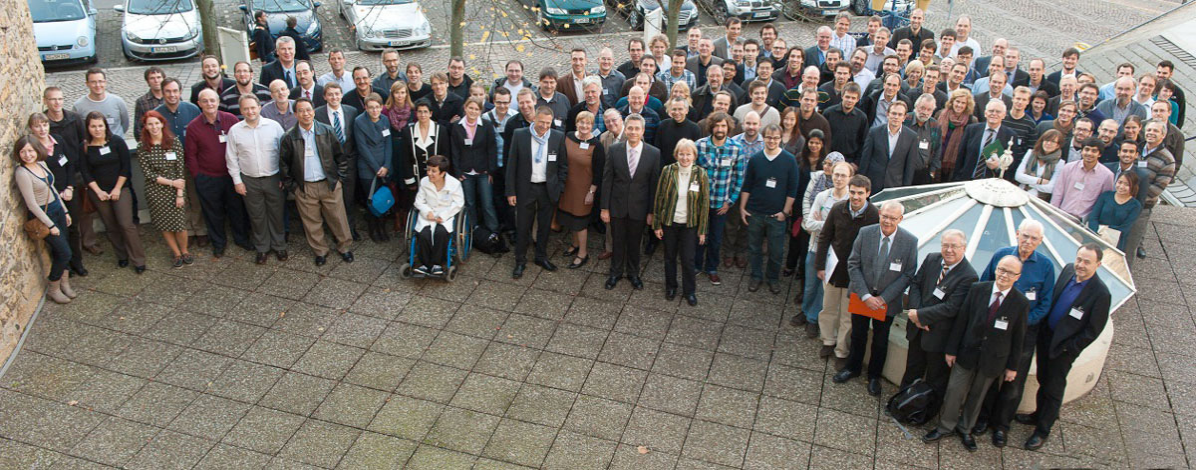
**Participants of the 8th German Conference on Chemoinformatics (GCC2012), November 11-13, 2012 in Goslar, Germany**.

Two different awards were bestowed for excellent scientific work at the GCC2012. Dr. Engelbert Zass from the ETH Zurich, Switzerland was presented with the Gmelin-Beilstein-Denkmünze [2] award for his plethora of contributions to numerous chemical informtation systems and databases over the past decades. Furthermore, the FIZ-CHEMIE-Berlin prizes for the best PhD thesis and the best diploma thesis in the field of Computational Chemistry were awarded to Dr. Anselm Horn from the University of Erlangen-Nürnberg, Germany and Florian Pfeiffer from the University of Stuttgart, Germany, respectively.

**Figure 2:**
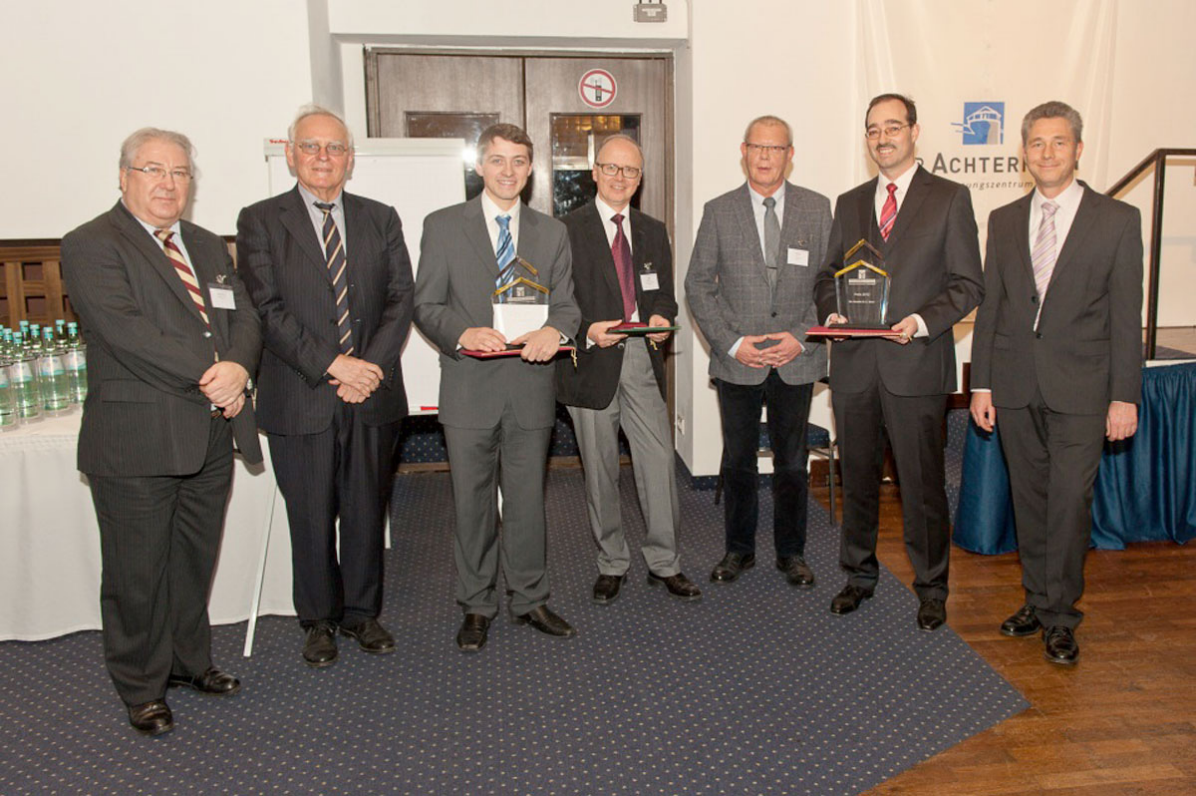
**FIZ CHEMIE Berlin Awards 2012 and GDCh Gmelin-Beilstein-Denkmünze Award: from left to right, Rene de Planque (General Secretary of the IUPAC), Henning Hopf (past president of the GDCh), Florian Pfeiffer (FIZ CHEMIE Berlin awardee, master thesis prize; University of Stuttgart, Germany), Engelbert Zass (Gmelin-Beilstein-Denkmünze Awardee; ETH Zurich, Switzerland), Jost Bohlen (FIZ CHEMIE Berlin), Anselm Holm (FIZ CHEMIE Berlin awardee, dissertation prize; University of Erlangen-Nürnberg, Germany) and Frank Oellien (Chair of the GDCh CIC division)**.
